# The Effect of Silanization Temperature and Time on the Marching Modulus of Silica-Filled Tire Tread Compounds

**DOI:** 10.3390/polym12010209

**Published:** 2020-01-15

**Authors:** Jungmin Jin, Jacques W. M. Noordermeer, Wilma K. Dierkes, Anke Blume

**Affiliations:** 1 Elastomer Technology and Engineering, Department of Mechanics of Solids, Surfaces and Systems (MS3), Faculty of Engineering Technology, University of Twente, 7500AE Enschede, The Netherlands; j.jin@utwente.nl (J.J.); j.w.m.noordermeer@utwente.nl (J.W.M.N.); a.blume@utwente.nl (A.B.); 2 HANKOOKTIRE Co., LTD. Main R&D Center, Material Department, Daejeon 34127, Korea

**Keywords:** marching modulus, silanization, silica compound, mixing, tire tread

## Abstract

Marching modulus phenomena are often observed in silica-reinforced solution styrene–butadiene rubber/butadiene rubber (S-SBR/BR) tire tread compounds. When such a situation happens, it is difficult to determine the optimum curing time, and as a consequence the physical properties of the rubber vulcanizates may vary. Previous studies have demonstrated that the curing behavior of silica compounds is related to the degree of silanization. For the present work, the effect of silanization temperature and time on the marching modulus of silica-filled rubber was evaluated. The correlations between these mixing parameters and their effect on the factors that have a strong relation with marching modulus intensity (MMI) were investigated: the amount of bound rubber, the filler flocculation rate (FFR), and the filler–polymer coupling rate (CR). The MMI was monitored by measuring the vulcanization rheograms using a rubber process analyzer (RPA) at small (approximately 7%) and large (approximately 42%) strain in order to discriminate the effects of filler–filler and filler–polymer interactions on the marching modulus of silica-filled rubber compounds. The results were interpreted via the correlation between these factors and their effect on the MMI. A higher temperature and a longer silanization time led to a better degree of silanization, in order of decreasing influence.

## 1. Introduction

Precipitated silica is widely used these days as reinforcing filler of tire tread compounds, since Michelin introduced the Green Tire technology in the 1990s [1]. Compared to a conventional filler such as carbon black, silica significantly improves tire performance. Many studies have proven the beneficial effect of using silica instead of carbon black from the perspective of rolling resistance, wet traction, and wear resistance [2,3,4,5,6,7]. However, the polarity difference between silica fillers and rubber polymers induces major difficulties in the mixing process such as high Mooney viscosities induced by poor dispersion. 

For the reinforcement of rubber, a high degree of filler dispersion is crucial. Additionally, the interaction between the filler and the rubber greatly contributes to the desired reinforcement induced by chemical or physical mechanisms. Therefore, a compatibilizer between silica and polymer—a silane coupling agent—is an essential component in the silica-filled rubber compound design. Via the silane coupling agent, silica and rubber are able to chemically interact during mixing as well as in the vulcanization process. Through the chemical reaction between silica and silane, which is commonly denoted as silanization, the polarity of silica greatly reduces and thus the processability and filler dispersion significantly improve [8,9,10]. After silanization, a further reaction between the silane and the rubber polymer occurs, which is denoted as filler–polymer coupling. The reinforcement therefore greatly improves, although the processability is negatively affected due to pre-scorch depending on the chemical structure of the silanes [11]. 

Mihara et al. [12] and Jin et al. [13] reported that the silanization temperature strongly affects the degree of silanization and the formation of chemically bound rubber. A too low temperature results in higher remaining filler–filler interaction and consequently in silica re-agglomeration. Reuvekamp et al. [11] reported that the mixing temperature and time is crucial in mixing silica and rubber with bis-(triethoxysilylpropyl)tetrasulfide (TESPT) as coupling agent in order to achieve a proper degree of silanization.

Because the silanization reaction must take place during the mixing cycle(s), many studies have been carried out to find the optimum mixing conditions for silica compounds. These studies are primarily focused on influences of mixer temperature settings, fill-factor, number of mixing stages, removal of ethanol generated during the silica-coupling agent reaction, etc. [14,15,16]. Besides these studies, many material developments for a better silica dispersion, such as the modification of polymers, silica, and silanes, are going on [17,18,19,20,21]. Overall, these studies primarily indicate that the silanization reaction plays a pivotal role for the dispersion and the bound rubber formation of a silica-filled rubber [22,23,24,25].

The consequence of an incomplete silanization is a marching modulus during the vulcanization stage of such compounds [13]. Marching modulus during vulcametry is a problem often observed in silica-filled solution styrene–butadiene rubber/butadiene rubber (S-SBR/BR) tire tread compounds. From an industrial standpoint, this phenomenon makes it difficult to determine the optimum curing time, t_c,90_ or t_c,95_, from a rheogram. It is neither productive nor efficient to wait for a prolonged time in order to obtain a plateau in the vulcanization profile. Therefore, the marching modulus phenomenon should be avoided or at least minimized. 

In order to overcome this problem, the effects of mixer silanization temperature and time (which are related to the degree of silanization) were evaluated and compared in the present work. Compounds with TESPT were mixed at various silanization temperatures and times. Subsequently, the vulcanization behavior of these compounds was measured at small (approximately 7%) and large (approximately 42%) strain. The results were interpreted in terms of the degrees of silanization reached, as well as the filler flocculation rate (FFR), filler–polymer coupling rate (CR), and the amount of bound rubber.

## 2. Materials and Methods

### 2.1. Materials and Mixing Procedures

**Figure 1:**
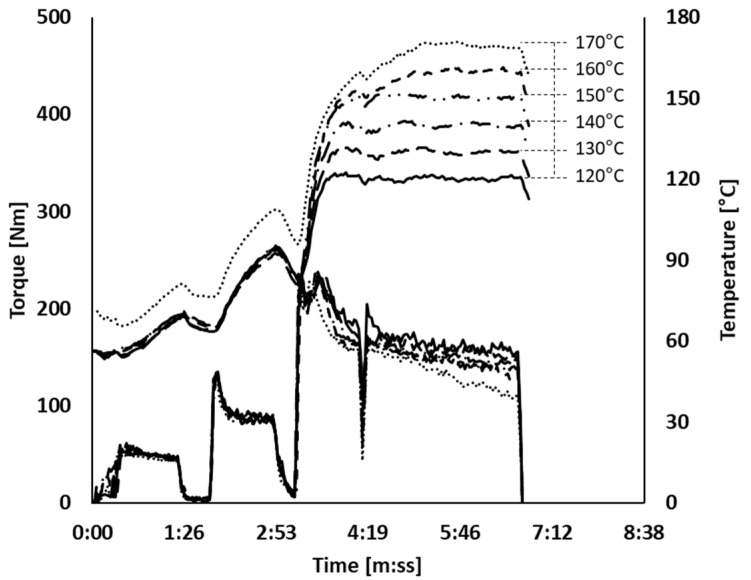
Mixing fingerprints of mixing procedure A; (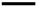): 120 °C; (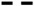): 130 °C; (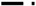): 140 °C; (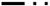): 150 °C; (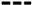): 160 °C; (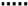): 170 °C.

**Figure 2:**
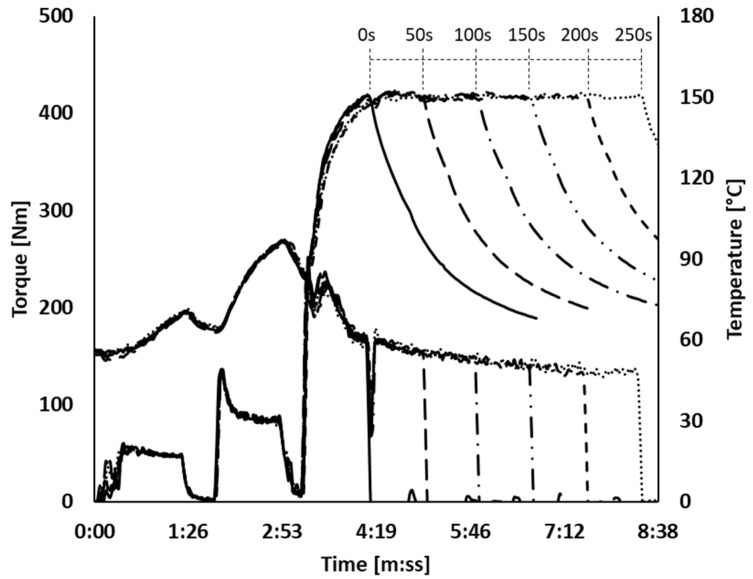
Mixing fingerprints of mixing procedure B; (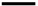): 0 s; (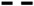): 50 s; (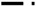): 100 s; (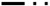): 150 s; (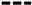): 200 s; (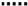): 250 s.

All series of experiments were done based on a tire tread compound as shown in Table 1. Two basic mixing procedures A and B were employed: procedure A to emphasize the effect of silanization temperature and procedure B to emphasize the effect of silanization time. The compounds were mixed in two steps as shown in Table 2 and Table 3. The master batch stage was done using a lab-scale internal mixer (Brabender Plasticorder, Brabender GmbH & Co KG, Duisburg, Germany) with 390 mL of chamber volume. The fill factor of the internal mixer was fixed to 63%. The temperature of the mixer temperature control unit (TCU) was set at 50 °C, except for the silanization temperature mixing test at 170 °C, where 70 °C of TCU temperature was applied for this test to reach 170 °C. In order to avoid the “first batch effect”, one initial batch was mixed and discarded before the regular mixing started. The regular mixing was started when the mixing chamber reached 55 °C. The mixing fingerprints are depicted in Figure 1 and Figure 2. In the case of mixing procedure A, the rotor speed was adjusted from 4:10 (minutes/seconds) onwards in order to reach and subsequently keep different silanization temperatures steady during a period of silanization of 150 s. The same temperature control technique was used for mixing procedure B, but here the silanization temperature was fixed at 150 °C and the silanization period varied. After the first mixing step, the compounds were sheeted out immediately on a lab-scale two-roll mill (Polymix 80 T, Servitec GmbH, Wustermark, Germany) in order to cool down the compounds and prevent further reaction. Three batches were mixed for each set of conditions in order to check the reproducibility. All batches were mixed with good reproducibility. 

The final mixing stage was done using a lab-scale two-roll mill (Polymix 80 T, Servitec GmbH, Wustermark, Germany). All ingredients to vulcanize the compound, alternatively called curatives (sulfur and curing accelerators such as zinc dibenzyldithiocarbamate (ZBEC) and *N*-cyclohexyl-2-benzothiazolesulphenamide (CBS)), were added in this step.

### 2.2. Filler–Filler Interaction Measured by Payne Effect

The Payne effect of silica-filled rubber compounds is generally used as an indicator of the degree of filler–filler interaction [22,26,27]. When a rubber is filled with a reinforcing filler, filler–filler interactions take place. In particular, silica fillers have many -OH groups on their surface and thus form a strong filler network in the rubber matrix via hydrogen bonding [28]. In general, the storage modulus of such filled rubber compounds decreases with increasing strain amplitude due to the breakdown of the filler network. This effect is commonly known as the Payne effect, which is obtained from the difference in storage moduli between low strain and high strain amplitude [26,27].

In the present work, the Payne effect values of the uncured rubber compounds were evaluated using a rubber process analyzer (RPA; RPA Elite, TA Instruments, New Castle, DE, USA). The storage shear moduli (G’) were measured at a temperature of 100 °C, a frequency of 0.5 Hz, and varying strains in the range of 0.56%–200%. The Payne effects were calculated from the difference in storage shear moduli at low strain (0.56%) and high strain (100%), that is, G’(0.56%)–G’(100%).

### 2.3. Filler Flocculation Rate

The filler flocculation rate (FFR) of the uncured silica-reinforced S-SBR/BR compounds without curatives was studied using the RPA mentioned above at 100 °C, a strain of 0.56%, and a test time of 14 min including 2 min of pre-heating time. The measurement temperature was selected according to a typical industrially employed extrusion temperature. The storage shear moduli were recorded at different measurement times. According to Mihara et al. [12], it is possible to observe the flocculation of silica particles by monitoring the change of storage modulus (G’) at low strain under isothermal conditions. The present results can best be fitted with Equation (1) or (2) [13,29]:

(1) $${G^{\prime }}_{0.56}\;\sim \;t^{FFR},$$

(2) FFR= dlog(G0.56′(t)G0,56i′)dlog(tti),

where *FFR* is a dimensionless flocculation rate, *G*′_0.56_(*t*) is the storage modulus at 0.56% strain at test time *t*; in Equation (2) normalized with *G*′_0.56*i*_, the initial storage modulus at *t*_i_, and *t*_i_ is 1 min.

### 2.4. Filler–Polymer Coupling Rate

The filler–polymer coupling rate (CR) of the uncured silica-reinforced S-SBR/BR compounds without curatives was studied using the RPA under the following conditions: 160 °C, 1.677 Hz, and 3° (~42% of strain) for 40 min. A large strain was applied for the CR measurement in order to break the filler–filler interaction. Therefore, only the filler–polymer interaction was taken into account in the CR. The torque levels at different times were recorded, and then the CR was calculated following Equation (3), based on the same concept as Equation (1) [13,29]:

(3) CR= dlog(T(t)Tscorch)dlog(ttscorch),

where *CR* is the dimensionless filler–polymer coupling rate, *T*(*t*) is the torque level at test time *t*, *T_scorch_* is the torque level at *t_scorch_*; the time to incipient cure or scorch which corresponds to the time for the torque to increase by 1 dNm is represented by *T*(*t*) = *T*_min_ + 1 dNm. *T*_min_ is the minimum torque level observed during the measurement.

### 2.5. Cure Characteristics and Marching Modulus Intensity

The rheograms were measured at 160 °C for 40 min under two different strain conditions as follows:

- 0.5° (~7% of strain) and 1.667 Hz of frequency: ASTM D5289-95 [30] conditions;
- 3° (~42% of strain) and 1.667 Hz of frequency [13].

The marching modulus intensity (MMI) was calculated from the rheograms using Equation (4) [13,29]:

(4) $$MMI=\;\frac{T_{40}-T_{20}}{40min-20min},$$

where *MMI* is the marching modulus intensity and *T*_40_ and *T*_20_ are the corresponding torques at 40 and 20 min.

### 2.6. Total Bound Rubber Content

Approximately 0.2 g of the rubber compounds without curatives, as obtained from the first mixing step, were cut into small pieces and immersed in toluene (technical grade toluene, Boom BV, Meppel, the Netherlands) at room temperature for 5 days, while the toluene was renewed every day. Thereafter, the samples were removed from the toluene, dried at 105 °C for 24 h, and weighed. The bound rubber content was calculated according to Equation (5) [31]:

(5) Bound rubber content (%)=Wfg−W(mf(mf+mp))W(mp(mf+mp))×100,

where *W_fg_* is the weight of filler plus gel, *W* is the original weight of the specimen, and *m_f_* and *m_p_* are the weights of filler and polymer in the compound, respectively.

### 2.7. Chemically and Physically Bound Rubber Content

The degree of filler–polymer coupling can be measured by the chemically bound rubber content. For this analysis, approximately 0.2 g of the rubber compounds without curatives, as obtained from the first mixing step, were cut into small pieces and immersed in toluene mentioned above at room temperature for 5 days, this time under ammonia (Ammonia 30% (as NH3) for analysis, ACS, PanReac Applichem GmbH, Darmstadt, Germany) atmosphere in order to cleave physical linkages. The toluene was renewed every day. Then, the samples were removed from the toluene, dried at 105 °C for 24 h, and weighed. The chemically bound rubber content was also calculated according to Equation (5). The physically bound rubber content was calculated by subtraction of the chemically bound rubber content from the total bound rubber content.

## 3. Results and Discussion

### 3.1. Filler–Filler Interaction (Payne Effect) as a Function of Silanization Temperature and Time

**Figure 3:**
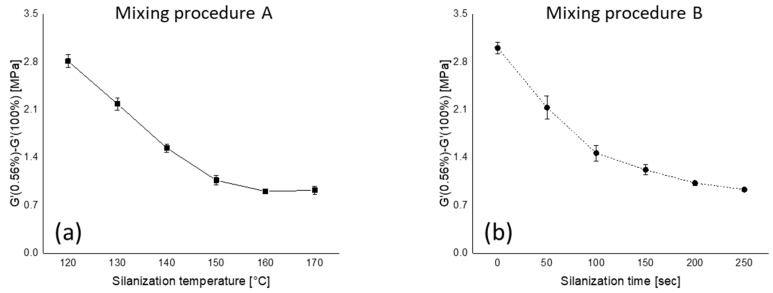
Payne effect as a function of (**a**) silanization temperature and (**b**) silanization time.

The Payne effect of the uncured compounds is plotted as a function of the silanization temperature and time for both mixing procedures, as shown in Figure 3. With increasing silanization temperature and time, a lower Payne effect value is observed due to a higher degree of silanization. 

As was already stated in our previous work, the mixing temperature greatly affects the degree of silanization [13]. Additionally, according to an Arrhenius plot, the reaction rate increases rapidly with temperature. Besides silanization temperature, the silanization time is also a major factor that enhances the degree of silanization. Even though the mixing temperature of all compounds in mixing procedure B reached 150 °C, a higher Payne effect value is observed when a relatively short or no silanization time was applied. The compound according to mixing procedure B with no silanization time shows even a 7% higher Payne effect level compared to the compound which was mixed at 120 °C according to mixing procedure A. These results indeed show that not only silanization temperature but also silanization time is crucial for the degree of silanization. However, the trend starts to level off when the silanization temperature and time reach 160 °C and 200 s, respectively. Therefore, a higher temperature than 160 °C and a duration longer than 200 s have little effect on reducing filler–filler interaction further.

### 3.2. Bound Rubber Content as a Function of Silanization Temperature and Time

**Figure 4:**
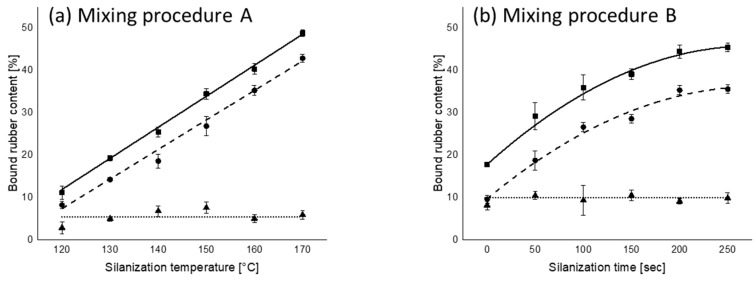
Bound rubber content as a function of (**a**) silanization temperature and (**b**) silanization time; (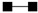): total bound rubber; (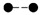): chemically bound rubber; (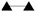): physically bound rubber.

Figure 4 shows the amount of bound rubber as a function of silanization temperature (mixing procedure A) and silanization time (mixing procedure B). Mixing condition (150 °C and 150 s) in both mixing procedures A and B was used as a reference silanization step. The bound rubber values corresponding to the reference silanization condition in Figure 4a,b show some differences. However, admittedly, the accuracy of the values of bound rubber tests is limited. When the actual values (Table 4) and the possible tolerance scale of the bound rubber evaluation (±5%) are taken into account, it can be stated that the values of the bound rubber corresponding to the reference silanization condition in Figure 4a,b are within the tolerance range. 

A higher amount of chemically bound rubber is observed when the compounds were mixed at a higher silanization temperature and a longer silanization time. It is well known that, already during mixing, some polymer–filler coupling via the silane may occur, in the worst case resulting in pre-scorch. Thus, a higher degree of polymer–filler coupling is already established during mixing at a higher silanization temperature or with a longer silanization time. For mixing procedure A, the amount of chemically bound rubber gradually increased along with silanization temperature, and a maximum value (~40%) was obtained at the highest silanization temperature employed, namely 170 °C. However, the maximum chemically bound rubber content (~35%) for mixing procedure B was observed after 200 s of silanization time and leveled off from this point onwards. This indicates that the silanization temperature is more effective for bound rubber formation than the silanization time. The physically bound rubber did not change, irrespective of the silanization conditions in either mixing procedure. 

**Figure 5:**
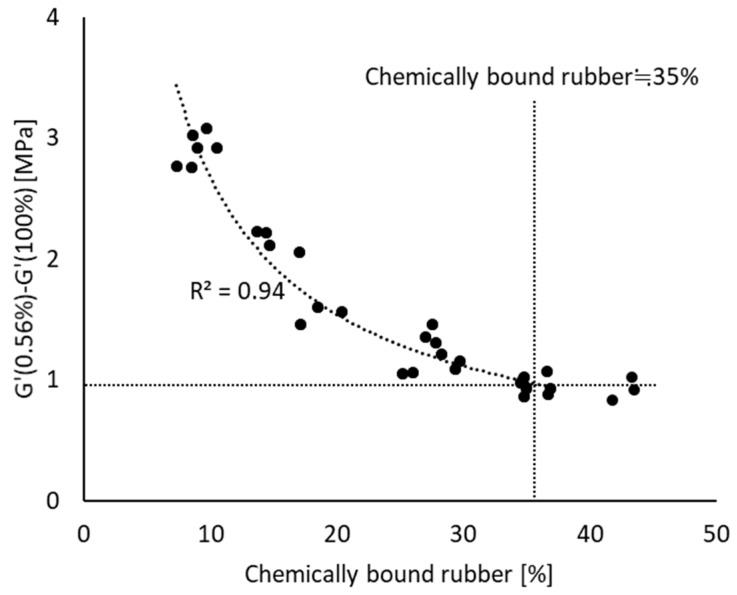
Payne effect vs. chemically bound rubber content; fitting line is based on polynomial fit with maximum *R*^2^.

All the Payne effect values of compounds mixed according to mixing procedures A and B and their chemically bound rubber content values show a good correlation, as shown in Figure 5, namely an *R*^2^ higher than 0.9. This indicates that the amount of chemically bound rubber strongly influences the reduction in filler–filler interaction. Another interesting point can be found in this figure. As was already stated based on Figure 3, from 160 °C onwards in mixing procedure A or 200 s in mixing procedure B, the Payne effects level off, and at these points both mixing procedures reach the same chemically bound rubber content of approximately 35%, see Figure 4. This indicates that a further reduction of filler–filler interaction with increasing chemically bound rubber content does not occur when the amount exceeds 35%.

### 3.3. Silica Filler Flocculation Rate

**Figure 6:**
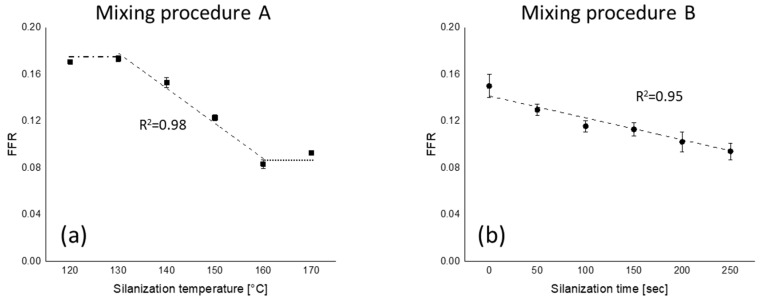
Filler flocculation rate (FFR) as a function of (**a**) silanization temperature and (**b**) silanization time; (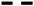): silanization temperature or time range where FFR shows good correlation with those parameters; (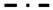): upper plateau; (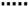): lower plateau; fitting lines for (**a**,**b**) are based on linear fit with maximum *R*^2^.

The silica filler flocculation rates (FFRs) are shown in Figure 6. The FFR values are plotted as a function of silanization temperature and time. A higher amount of bound rubber leads to lower filler–filler interaction and thus a reduced tendency for flocculation. However, the maximum difference of FFR for mixing procedure B is smaller than for mixing procedure A due to a lower amount of total and chemically bound rubber. 

In our previous work, it was already reported that the FFR has an upper limit [13]. However, in the present work, mixing procedure A shows two plateau regions—one for a silanization temperature lower than 130 °C and one for temperatures higher than 160 °C. This indicates that the FFR has an upper and lower limit; in this compound, an upper limit of 0.17 and a lower limit of 0.09 was found. In the case of mixing procedure B, no upper and lower limits are observed because the range of FFR values was within those limits of FFR. 

**Figure 7:**
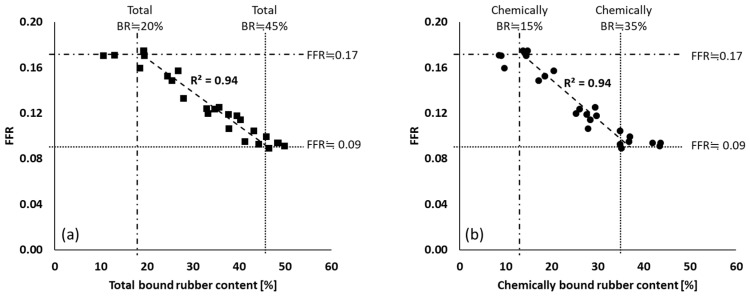
FFR vs. bound rubber content: (**a**) FFR vs. total bound rubber and (**b**) FFR vs. chemically bound rubber; (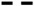): silanization temperature or time range where FFR shows good correlation with those parameters; (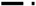): upper limit; (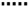): lower limit; fitting lines for (**a**,**b**) are based on linear fit with maximum *R*^2^.

FFR values of all mixing series are plotted against total and chemically bound rubber content of these compounds in Figure 7. The FFR values start to decrease when the total and chemically bound rubber contents exceed approximately 20% and 15%, respectively. No correlation was observed between FFR and physically bound rubber. This means that it is only the chemically bound rubber that strongly influences the silica flocculation behavior, and thus the degree of silanization is the key for FFR reduction. 

### 3.4. Filler–Polymer Coupling Rate after Mixing

**Figure 8:**
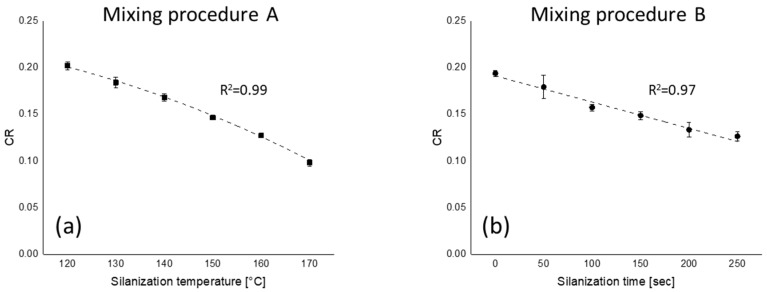
Coupling rate (CR) as a function of (**a**) silanization temperature and (**b**) silanization time; fitting lines for (**a**) and (**b**) are based on polynomial and linear fit with maximum *R*^2^, respectively.

The filler–polymer coupling rates (CRs) of all mixing series are shown in Figure 8. The CRs of the compounds plotted as a function of the silanization temperature and time show a decreasing trend with temperature and time. Similar to the FFR results, the difference of CR for mixing procedure B is smaller than for mixing procedure A—silanization temperature has more effect on the filler–polymer coupling reaction than silanization time. 

**Figure 9:**
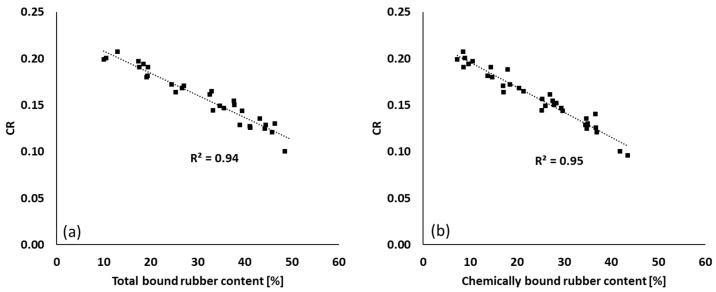
CR vs. bound rubber content: (**a**) CR vs. total bound rubber and (**b**) CR vs. chemically bound rubber; fitting lines for (**a**,**b**) are based on linear fit with maximum *R*^2^.

The CRs of all mixing series are plotted as a function of total and chemically bound rubber contents of these compounds in Figure 9. Both types of bound rubber show a good correlation with CR. As was already reported, at a higher silanization temperature during mixing, the coupling agent TESPT releases more active or free sulfur and begins to give some filler–polymer bonding [13,32]. As a result, the concentration of free sulfur as well as the amount of leftover silane decreases when mixing is done at a higher temperature. Consequently, the CR decreases due to the lowered concentration of active sulfur and remaining free silane. 

### 3.5. Marching Modulus Intensity

**Figure 10:**
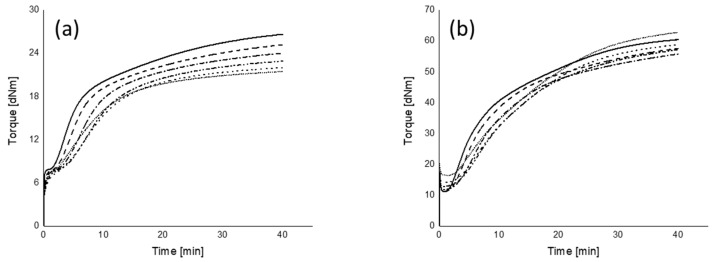
Rheograms of mixing procedure A: (**a**) 0.5° (~7% of strain) and (**b**) 3° (~42% of strain); (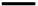): 120 °C; (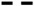): 130 °C; (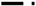): 140 °C; (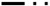): 150 °C; (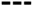): 160 °C; (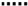): 170 °C.

**Figure 11:**
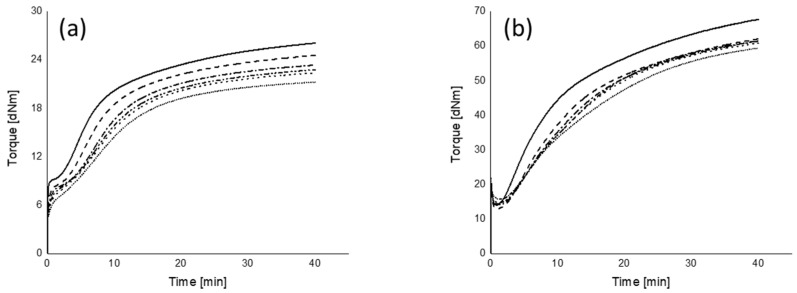
Rheograms of mixing procedure B: (**a**) 0.5° (~7% of strain) and (**b**) 3° (~42% of strain); (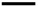): 0 s; (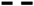): 50 s; (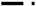): 100 s; (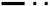): 150 s; (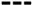): 200 s; (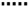): 250 s.

**Figure 12:**
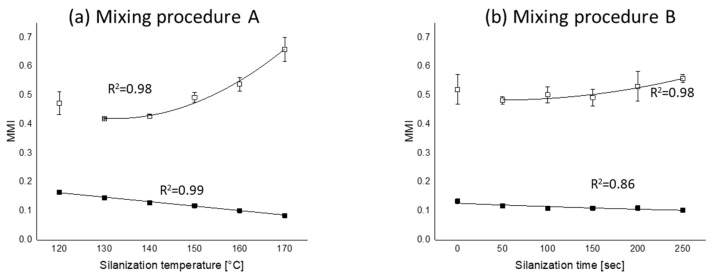
Marching modulus intensity (MMI) as a function of silanization temperature and time: (**a**) MMI vs. silanization temperatures and (**b**) MMI vs. silanization time; (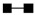): MMI 0.5°; (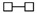): MMI 3°; fitting lines for each MMI in (**a**,**b**) are based on linear or polynomial fit with maximum *R*^2^.

The rheograms of the mixing procedures A and B and their marching modulus intensities (MMIs) of all mixing series measured at two different strains are plotted in Figure 10, Figure 11 and Figure 12, respectively. 

#### 3.5.1. Marching Modulus Intensity at Small (ASTM Conditions, MMI 0.5°) and Large Strain (MMI 3°)

As can be seen in Figure 10, Figure 11 and Figure 12, the MMI 0.5° decreases with silanization temperature and time; however, silanization time shows a slightly lower correlation coefficient. The maximum difference of MMI 0.5° of the compounds in mixing procedure B is about 40% of mixing procedure A, and the MMI values in the range of 50 to 200 s of silanization time are almost the same. The MMI at 3° plotted as a function of both silanization temperature and time, as shown in Figure 12, show good correlations, except for the compounds mixed at a very low silanization temperature of 120 °C or silanization time of 0 s. These exceptional MMI 3° values can be explained by the intensity of filler–filler interaction still present at the early stage of silanization, as seen in Figure 3 with the high Payne effects.

Different from the trend of MMI 0.5°, the MMI 3° increases with silanization temperature and time, see Figure 12. The reason is the decreasing vulcanization speed, as observed in Figure 10b and Figure 11b, for all mixes with increasing silanization temperature and time, even though they reach approximately the same end level with the exception of the curves for the silanization temperature of 170 °C in Figure 10b and the silanization time of 0 s in Figure 11b. 

#### 3.5.2. Marching Modulus Intensity as a Function of FFR and CR

**Figure 13:**
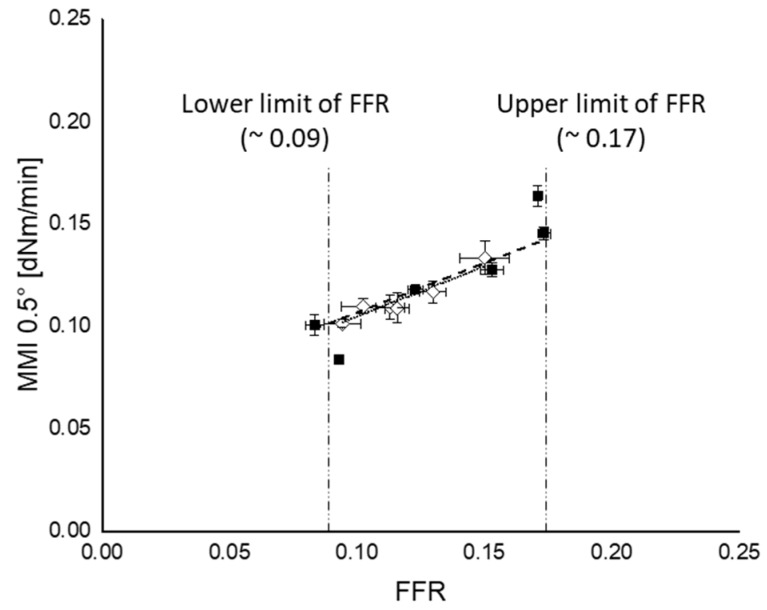
MMI 0.5° vs. FFR; (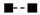): mixing procedure A; (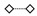): mixing procedure B.

**Figure 14:**
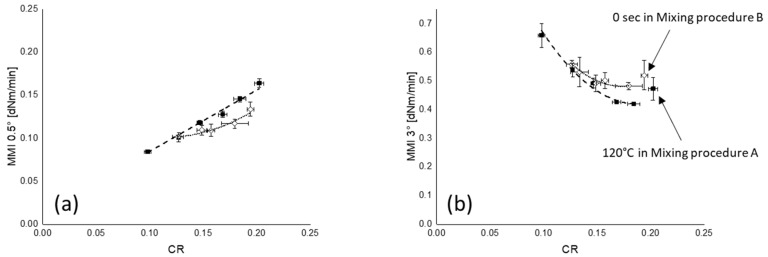
MMI vs. CR: (**a**) MMI 0.5° and (**b**) MMI 3°; (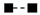): mixing procedure A; (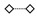): mixing procedure B.

A correlation was made between the MMI and the FFR and CR as shown in Figure 13 and Figure 14, respectively. Within the range of lower and upper limit of FFR, both mixing procedures A and B show good correlations with silanization temperature and time, namely *R*^2^ = 0.96 and 0.91, respectively. Since the influence of filler–filler interaction becomes less for MMI 3° due to the high deformation strain, no correlation could be made between FFR and MMI 3° and is therefore not depicted in Figure 13. 

Not only the FFR but also the CR is important for the MMI 0.5° and MMI 3°. In Figure 14, both MMIs are plotted as a function of CR. A higher silanization temperature and a longer silanization time lead to a higher amount of bound rubber and indicate a lower concentration of free silane and released free sulfur from TESPT. As a consequence, a slower and lower degree of the coupling reaction occurs during the vulcanization process. Finally, a lower torque level and corresponding flat plateau of the rheograms is obtained when the rheometer runs at small strains, approximately 7% (~0.5°). In the case of large strain, approximately 42% (~3°), a rapid rise of the rheometer torque can be seen in the compound, which was mixed at a lower silanization temperature or a shorter silanization time, due to a faster filler–polymer coupling reaction; a relatively large amount free silane and free sulfur remains within the compound. These results again show that the shielding of the filler surface not only by a silane coupling agent but also by bound rubber does strongly affect the rheometer torque.

## 4. Conclusions

The focus of this study was to investigate mixing parameters that can reduce the marching modulus in a rheometer curve during the curing of silica-filled S-SBR/BR rubber compounds. The silanization temperature and time strongly influence the marching modulus phenomenon. A higher temperature and a longer time for the silanization lead to a better degree of silanization, where temperature has the largest effect. 

The amount of chemically bound rubber of a silica compound has an intricate influence on the filler–filler interaction as well as on the silica flocculation rate and the filler–polymer coupling rate, all strongly related to the marching modulus. The chemically bound rubber suppresses the flocculation behavior of the silica in rubber during the vulcanization process. However, a very low or even very high amount of chemically bound rubber no longer affects the silica flocculation rate, indicating that the FFR has an upper and a lower limit. The consequence of a higher amount of chemically bound rubber is a lower concentration of free silane which may enhance the filler–polymer coupling reaction during vulcanization. Therefore, as the amount of bound rubber increases, a slower filler–polymer coupling rate is obtained during vulcanization and results in a reduction of the marching modulus phenomenon of silica compounds.

## Figures and Tables

**Table 1:** Compound formulation. phr, xxxxx.

Mixing Stage	Ingredient	Product Name	Company	Content (phr ^8^)
Master batch	S-SBR ^1,2^	VSL5025-2HM	Lanxess (Cologne, Germany)	110
	BR ^3^	BUNA CB24	Lanxess (Cologne, Germany)	20
	Silica	ULTRASIL7005 (CTAB:164 m^2^/g)	Evonik (Wesseling, Germany)	90
	Silane (TESPT)	Si69	Evonik (Wesseling, Germany)	8.1
	TDAE oil ^4^	Vivatec 500	Hansen & Rosenthal (Hamburg, Germany)	5
	Stearic acid	Stearic acid	Merck (Darmstadt, Germany)	1
	Zinc oxide	ZnO	Merck (Darmstadt, Germany)	2
	DPG ^5^	Perkacit DPG	Flexsys (Deventer, Netherland)	1.5
Final	Sulfur	S	J. T. Baker (Landsmeer, Netherlands)	0.7
	ZBEC ^6^	Vulkacit ZBEC	Lanxess (Cologne, Germany)	0.2
	CBS ^7^	Santocure CBS	Flexsys (Deventer, Netherland)	2.2

^1^ S-SBR, solution styrene–butadiene rubber; ^2^ 27.3 wt % TDAE oil extended.; ^3^ BR, butadiene rubber; ^4^ TDAE oil, treated distillate aromatic extract oil; ^5^ DPG, 1,3-diphenylguanidine; ^6^ ZBEC, zinc dibenzyldithiocarbamate; ^7^ CBS, *N*-cyclohexyl-2-benzothiazolesulphenamide; ^8^ phr, parts per hundred rubber.

**Table 2:** Master batch mixing procedure.

Mixing Procedure A		Mixing Procedure B	
Action	time (mm:ss)	Action	time (mm:ss)
Add polymer	00:00 to 00:20	Add polymer	00:00 to 00:20
Mastication	00:20 to 01:20	Mastication	00:20 to 01:20
½ Silica, Silane	01:20 to 01:40	½ Silica, Silane	01:20 to 01:40
Mixing	01:40 to 02:40	Mixing	01:40 to 02:40
½ Silica, remaining ingredients	02:40 to 03:10	½ Silica, remaining ingredients	02:40 to 03:10
Mixing till target temperature: 120, 130, ...., 170 °C	03:10 to 04:10	Mixing till 150 °C	03:10 to 04:10
Ram sweep	04:10 to 04:14	Ram sweep	04:10 to 04:14
Mixing (at target temperature)	04:14 to 06:40	Mixing for various time laps: 0, 50, 100, …., 250 s	04:14 to 08:20
Discharge and sheeting	-	Discharge and sheeting	-

**Table 3:** Final mixing procedure for A as well as for B.

Action	Time (mm:ss)
Add master batch	-
Mixing	00:00 to 02:00
Add curatives	02:00 to 02:30
Mixing	02:30 to 09:00
Discharge	

**Table 4:** Amount of bound rubber of mixing procedures A and B corresponding to the reference silanization condition (150 °C and 150 s).

Bound Rubbers	Mixing Procedure A	Mixing Procedure B
Chemically	27%	29%
Physically	8%	11%
Total	35%	40%
